# The Association between Gender and Physical Activity Was Partially Mediated by Social Network Size during COVID-19

**DOI:** 10.3390/ijerph19052495

**Published:** 2022-02-22

**Authors:** Ashley Kuzmik, Yin Liu, Yendelela Cuffee, Lan Kong, Christopher N. Sciamanna, Liza S. Rovniak

**Affiliations:** 1 College of Nursing, Pennsylvania State University, 306 Nursing Sciences Building, University Park, Philadelphia, PA 16802, USA; 2 Department of Human Development and Family Sciences, Utah State University, 2905 Old Main Hill, Logan, UT 84322, USA; yin.liu@usu.edu; 3 College of Health Sciences, University of Delaware, 100 Discovery Boulevard, Newark, DE 19713, USA; ylcuffee@udel.edu; 4 Departments of Medicine and Public Health Sciences, College of Medicine, Pennsylvania State University, Hershey, PA 17033, USA; luk14@psu.edu (L.K.); csciamanna@pennstatehealth.psu.edu (C.N.S.); lrovniak@pennstatehealth.psu.edu (L.S.R.)

**Keywords:** COVID-19, women’s health, physical activity, social networks

## Abstract

The COVID-19 pandemic has disrupted physical activity, particularly among women. Limited research has explored how social network support may explain gender-based variations in physical activity during COVID-19. The purpose of this study was to examine the mediating role of social networks in the association between gender and physical activity during a pandemic. This cross-sectional survey assessed whether social network characteristics (i.e., in-person social network size, frequency of in-person social network interactions, and online friend network size) mediate the relationship between gender and either past-week or past-year physical activity. Multiple mediation analyses were conducted to determine the indirect effect of gender on physical activity through social networks. Among 205 participants, women (n = 129) were significantly less physically active (β = −73.82; *p* = 0.02) than men (n = 76) and reported significantly more Facebook friends (β = 0.30; *p* < 0.001) than men, which was inversely associated with past-week physical activity (β = −64.49; *p* = 0.03). Additionally, the indirect effect of gender on past-week physical activity through Facebook friends was significant (β = −19.13; 95% CI [−40.45, −2.09]). Findings suggest that social media sites such as Facebook could be used to encourage physical activity among women during a pandemic.

## 1. Introduction

The Physical Activity Guidelines for Americans recommend that adults 18 years or older perform at least 150 min per week of moderate-intensity exercise or 75 min per week of vigorous-intensity exercise along with at least two days of muscle-strengthening activity to achieve the health benefits associated with an active lifestyle [1]. Women consistently fall behind their male counterparts in their quantity of moderate and vigorous physical activity [2,3]. Pre-COVID-19, a study across 168 countries with almost 2 million participants (≥18 years of age) revealed that 32% of women versus 23% of men are inactive [4].

Since the onset of the COVID-19 pandemic, women have continued to be less engaged in physical activity than men [5,6]. These gender differences in physical activity have largely been attributed to pre-existing low levels of social support [5,7] and heightened demands, such as managing full-time employment together with household tasks and child care [7]. Alongside women’s lower levels of activity, COVID-19 restrictions that increase social isolation have exacerbated concerns associated with mental health [7,8,9]. Specifically, relative to men, women with less physical activity experienced greater mental health concerns (e.g., anxiety) during the pandemic [5]. Women’s lower physical activity levels have also been linked to increased risk, relative to men, of chronic health conditions, such as cardiovascular disease, cancer and type 2 diabetes [10,11].

Although time constraints and low motivation are common physical activity barriers among women [12], social network support for engaging in physical activity can contribute to reducing these time-related and motivational barriers [13]. The multilevel Social Networks for Activity Promotion (SNAP) model [14] is a framework that demonstrates how physical activity can be influenced by social networks at three different levels: (1) built physical environment (i.e., built, natural, or online settings); (2) social network environment (i.e., population attributes and interaction modes); and (3) social network interactions (social network structure and functions). The SNAP model indicates that both in-person and virtual/online environments may provide either social network support or constraints, which can function, respectively, to promote or inhibit social interactions related to physical activity. Research indicates that greater social network support can influence past-week and past-year physical activity [15].

Previous studies have predominantly emphasized descriptive differences between women’s and men’s social network structure, defined as the number and frequency of contacts with others who may support or constrain efforts to engage in physical activity [14]. For instance, in pre-pandemic studies investigating social network structure, women tended to have a larger number of networks with a greater diversity of connections (e.g., family, friends, and neighbors), whereas men often had a smaller network size with fewer connections (e.g., co-workers) [16,17,18]. Additionally, women have more online friends compared to men [19,20], evident in social network sites such as Facebook [20]. To promote physical activity among women during a pandemic, and other periods when physical activity may be particularly compromised, it is important to understand whether modifiable social network characteristics mediate the relationship between gender and physical activity. Such an understanding would inform the design of future in-person and online physical activity interventions.

Although evidence of pre-pandemic associations between gender, social networks, and physical activity exists [14,17,18,20], prior research has not investigated social network characteristics as a mediating pathway linking gender with physical activity. To address this gap, the purpose of this study was to investigate the process by which in-person and online social network structure explains gender-based differences in physical activity. To capture dimensions of social network structure that could be efficiently assessed and confirmed in other independent samples, we used validated self-report measures of social network structure that were described previously [21]. Further, because levels of physical activity fluctuate, both past-year and past-week total minutes were considered to provide a more comprehensive estimate of typical activity levels than either measure alone. Based on prior research [14,17,18,20], we hypothesized that the association between gender and physical activity would be mediated by social network structure.

## 2. Materials and Methods

### 2.1. Study Design

This cross-sectional study was conducted between November 2020 through February 2021, as part of a larger measurement validation study. Ethical approval was obtained by the Pennsylvania State University College of Medicine Institutional Review Board (STUDY00000055). Participants provided informed consent via an online form.

### 2.2. Sample

Participants were required to be: (1) age 18 years or older; (2) comfortable answering written questions in English; (3) “highly physically active”, i.e., engage in 150 min/week or more of regular moderate-vigorous physical activity for at least one year and at least two different modes (types) of physical activity (i.e., strength training and at least one other physical activity mode, such as walking/running), or “highly physically inactive” (60 min/week or less of regular moderate-vigorous physical activity for at least one year) based on three brief validated questionnaires [22,23,24,25]; (4) residing current address for at least 6 months; and (6) able to provide a valid Amazon Turk Worker ID for future contact.

Exclusion criteria were: (1) pregnancy; (2) inability to walk three city blocks or another medical condition limiting physical activity participation; (3) residence in an institutionalized setting (e.g., retirement home, hospital, prison, mental health facility, etc.) or homeless; (4) plan to relocate within the next month; and (5) provision of Amazon Turk Worker ID that duplicated one received previously. Additionally, participants were recruited from the “most” active US states and from the “least” active US states, based on the proportion of adults in each state meeting aerobic and muscle-strengthening guidelines between 2011 and 2019, as determined by the Behavioral Risk Factor Surveillance System [26]. The “most” active states included Colorado, Hawaii, District of Columbia (DC), Alaska, California, New Mexico, Utah, Vermont, Washington, Wyoming, and Connecticut. The “least” active states included West Virginia, Mississippi, Arkansas, Alabama, Tennessee, Oklahoma, Kentucky, Louisiana, North Dakota, Indiana, and Missouri.

### 2.3. Study Procedures

Participants were recruited through CloudResearch (https://www.cloudresearch.com accessed on 28 February 2021)—an online research platform linked with Amazon Mechanical Turk (MTurk). Specifically, MTurk is an online crowdsourcing platform where researchers post a job assignment called a HIT (Human Intelligence Task), and eligible MTurk workers (≥18 years of age) have an opportunity to complete the HIT for compensation. The HIT includes a title and description of the job assignment. It also displays the amount of compensation and time it takes to complete the task. MTurk data exhibit evidence of validity and reliability across various tasks and populations [27,28,29].

In our study, interested qualified MTurk workers were directed to a study link provided as part of the job assignment (i.e., HIT) that was posted on MTurk’s website to be completed by workers for pay. MTurk workers were eligible to participate if they had a minimum 90% approval rating and at least 100 HITS completed. The study link contained a brief header to describe the study. After reading the online consent information, participants were invited to provide implied informed consent online. Those providing consent were directed to complete the screening form (~5–10 min) through a REDCap survey link [30]. If participants remained eligible after completing the screening form, they were asked to complete an online social environment questionnaire (~20 min) and were emailed a REDCap link to this questionnaire using their worker ID within 24 h of determining eligibility. Participants had up to seven days to complete the questionnaire, and a maximum of three email reminders were sent. Participants were paid $0.50 after completing the screening form, and if eligible, $1.50 for completing the social environment questionnaire. A unique code was provided to participants to copy and paste into the CloudResearch platform to verify survey completion and obtain compensation for their time.

Attention check questions (blinded to respondents) were included [31]: (1) age in the screening form and year born in survey 1 (should not have more than 1 year difference across measures); and (2) worker ID across all surveys (should be identical across all measures). These attention check questions were naturally embedded in the surveys to validate responses [31] and to differentiate between high-quality and low-quality (inconsistent) responses. A syntax program was written in SPSS to automatically scan all the surveys for responses on the attention check questions (Supplementary Syntax S1). Participants who failed any of the attention check questions were excluded from analyses.

### 2.4. Measures

#### 2.4.1. Independent Variable

The independent variable was gender, a binary variable, with “male” coded as 0 and “female” coded as 1.

#### 2.4.2. Dependent Variables

##### Past-Week Physical Activity

The annual National Health Interview Survey (NHIS) is administered by the Centers for Disease Control and Prevention’s (CDC) National Center for Health Statistics (NCHS) [22] and evaluates various health topics, including physical activity. The NHIS provides a reliable and valid report of moderate-vigorous physical activity and strength training activity over one week [22,32]. The outcome variable used in analyses was the total number of minutes of moderate-vigorous activity performed over one week.

##### Past-Year Physical Activity

The Chasan-Taber Physical Activity Questionnaire (CT-PAQ) is a self-report questionnaire that measures the duration, frequency, and intensity of lifetime recreational physical activities [23,24]. In this study, a subset of this questionnaire was used to evaluate participants’ past-year physical activity. The CT-PAQ is highly reliable [23] with concurrent validity between the questionnaire-based activity scores and physical activity log scores [24]. The outcome variable used in the analyses was the number of minutes of moderate-vigorous activity performed over one year.

#### 2.4.3. Mediator Variables

##### Social Network Size and Frequency of Contact

Social network size and frequency of social contact were measured using the Berkman–Syme Social Network Index (SNI) [21], which has demonstrated adequate test–retest reliability (alpha = 0.64–0.70) and construct validity [33,34]. Respondents selected “None”, “1 or 2”, “3–5”, “6–9”, or “10 or more” to characterize their social network size, that is, the number of close friends and relatives. These items were subsequently scored as ≤2 friends or ≤2 relatives = 0 and all other scores = 1, following previously used procedures [35]. Frequency of social contact (the frequency of monthly contact with close friends or relatives) was similarly scored as ≤2 friends or ≤2 relatives = 0 and all other scores = 1 [35].

##### Number of Facebook Friends

Facebook friends number was dichotomized using the standard median split approach [36,37], based on evidence that “low” (coded “0”) and “high” (coded “1”) numbers of friends are associated with differential health outcomes [38,39].

#### 2.4.4. Control Variables

The analyses controlled for demographic covariates, including race/ethnicity, education, and high versus low active states. These variables have been previously found to co-vary with gender and physical activity [4,26,40]. The COVID-19 seven-day case rate per 100,000 people was included as a continuous covariate to consider the differential impact of pandemic severity across the states measured [41].

### 2.5. Data Analysis

Statistical analyses were performed using SPSS (IBM Corporation, Armonk, NY, USA) and AMOS versions 27 [42] with significance levels at an alpha level of 0.05. Descriptive statistics were used to assess sample characteristics. Normality of the variables was verified using the Shapiro–Wilk test. Associations between study variables were assessed using Pearson’s correlation and presented in a heatmap. Highly correlated variables (r > 0.90) indicating multicollinearity and variables that were not correlated with either the independent variable (gender) or dependent variables (past-week or past-year physical activity) were excluded from the mediation analyses [43].

**Figure 1:**
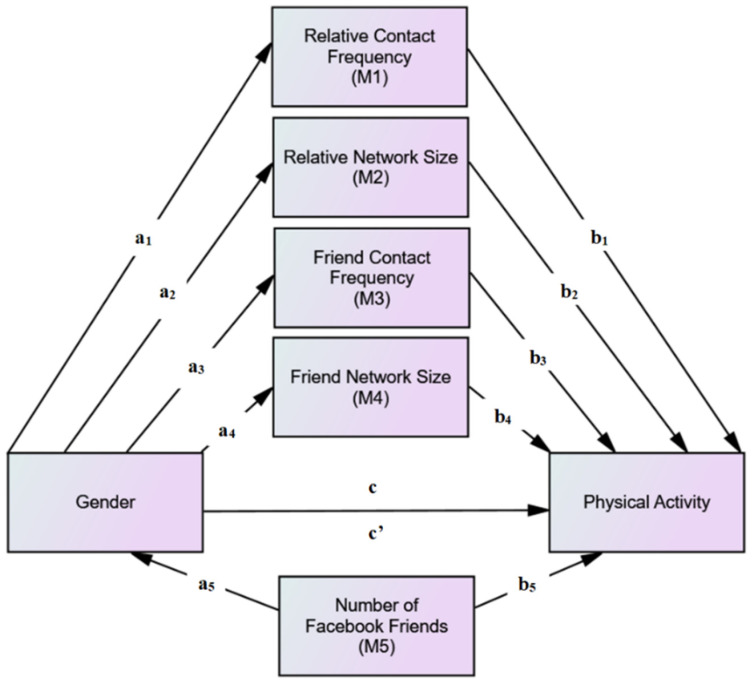
Path diagram of multiple mediation model testing combined effects of proposed mediators of the relationship between gender and physical activity. Paths A: Represent association between gender and mediator variables. Paths B: Represent association between mediator variables and physical activity. Paths C: Represent whether gender and mediator variables together are predictors of physical activity.

Based on the steps proposed by Preacher and Hayes [44], multiple mediation analyses were conducted (see Figure 1) to assess whether social network characteristics mediate the relationship between gender (independent variable) and physical activity (dependent variable; past-week and past-year minutes of physical activity). To test for the mediation effects, two multiple mediation models were performed for each outcome, applying five mediators for each single analysis. Path C represented the main effect of gender as a predictor of physical activity. The A paths (a_1_–a_5_) represented the association between gender and the mediator variables (i.e., social network size and frequency of social network interactions). The B paths (b_1_–b_5_) represented the association between the mediator variables and physical activity. Lastly, for path C, both gender and mediator variables were entered simultaneously as predictors of physical activity. The following conditions were required to establish mediation: (1) the magnitude of association was statistically significant in A and B paths, and (2) the level of significance of the coefficient in path C was either non-significant (full mediation) or less significant, in which case the total effect between the independent and dependent variable still exists, but in a smaller magnitude (partial mediation). A bootstrap sampling procedure assessed the significance of the total and specific indirect effects for each mediator to estimate mediation [44,45,46]. Bootstrap estimates were based on 5000 bootstrap samples. The indirect effect was considered statistically significant on the condition that the 95% confidence interval did not include zero. All mediation analyses controlled for race/ethnicity, education, and state-based physical activity level. Age and marital status were not significantly associated with gender, social networks, or physical activity (*p* > 0.05, respectively), and thus, were not controlled for in the model.

## 3. Results

### Study Characteristics

As shown in Table 1, most of the sample (N = 205) was female (62.93%), non-Hispanic (96.59%), white (85.36%), and had a mean age of 40.98 ± 12.89 years. Participants reported a range of self-rated health statuses, including excellent health (18.54%), very good health (35.61%), fair health (14.63%), and poor health (1.46%). Among the participants, 46.83% were married, 35.63% had a 4-year college degree, 25.85% earned an income between $30,000 and $49,999, and 75.12% lived in a single-family house. The mean number of Facebook friends was 255.81 ± 375.37. Participants spent on average 168.79 ± 220.23 min in moderate-vigorous physical activity per week and 232.27 ± 350.64 min (per week) in moderate-vigorous activity over the past year.

**Figure 2:**
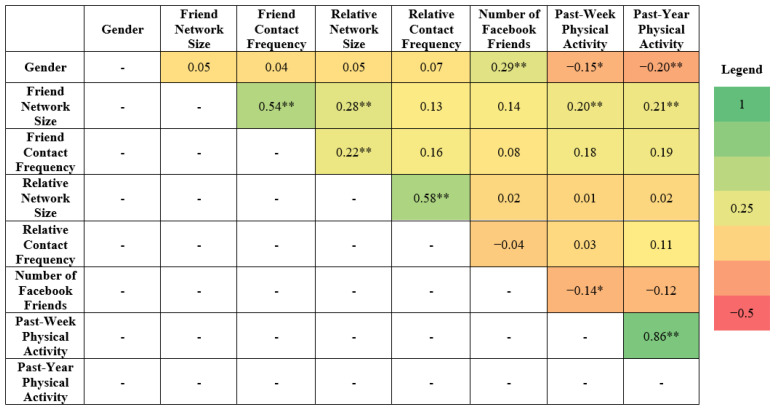
Heatmap of Pearson correlation coefficients between the study variables. Notes: * *p* ≤ 0.05; ** *p* ≤ 0.01.

Figure 2 shows that the highest correlation among the predictor variables was 0.58, suggesting that the shared variance between each variable was sufficiently small that each variable could contribute to the overall analysis. Relative network size and relative contact frequency were not associated with gender or physical activity and therefore excluded as mediators from the models. Additionally, Facebook friends number was not associated with past-year physical activity and was not included in the mediation analysis.

**Figure 3:**
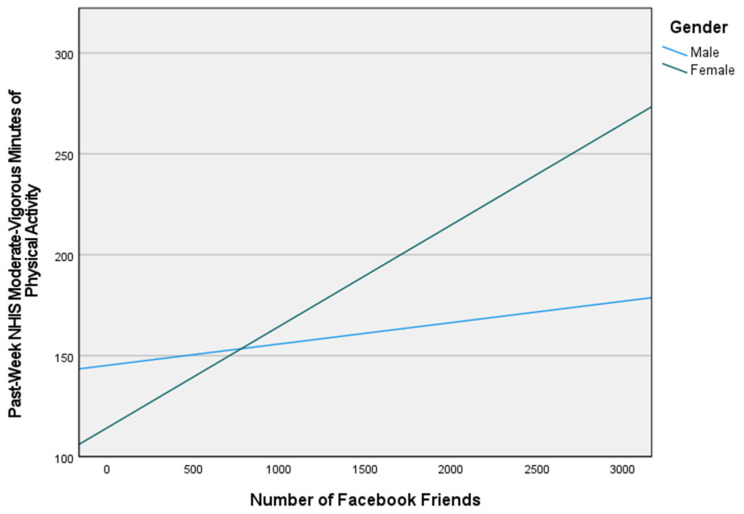
Graphic representation of the number of Facebook friends by gender on NHIS moderate-vigorous minutes of physical activity. Notes: NHIS Moderate-Vigorous Physical Activity.

Figure 3 shows that women with more Facebook friends perform more minutes of past-week physical activity compared to men. For example, a woman with 1800 Facebook friends performed 38.33 more minutes of physical activity per week, on average, than a man with 1800 Facebook friends.

**Figure 4:**
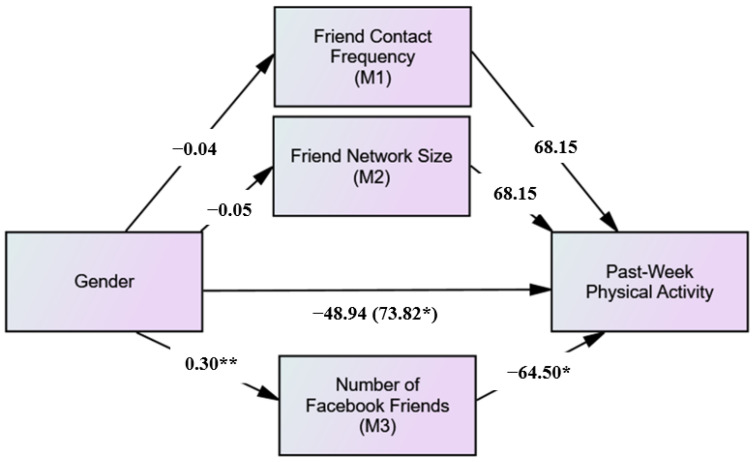
Mediation effects of number of Facebook friends on the relationship between gender and past-week physical activity. Notes: * *p* < 0.05, ** *p* < 0.001; Total effect (C path) in parentheses.

As shown in Figure 4, the results of the mediation analysis revealed there was a significant total effect of gender on past-week physical activity (β = −73.82; *p* = 0.02), and when Facebook friends number (mediating variable) was added into the model, the association between gender and past-week physical activity became non-significant (β = −48.94; *p* = 0.12). The model revealed that women had significantly more Facebook friends than men (β = 0.30; *p* < 0.001), and Facebook friends number was significantly and inversely related to past-week physical activity (β = −64.49; *p* = 0.03). Furthermore, as shown in Table 2, the indirect effect of Facebook friends number between gender and past-week physical activity was significant, but the indirect effects were not significant for friend network size and friend contact frequency. No significant indirect effects were found for friend network size and friend contact frequency between gender and past-year physical activity.

## 4. Discussion

The purpose of this study was to examine the contribution of in-person and online social network structure during COVID-19 to explain gender-based disparities in physical activity. Although previous studies have descriptively summarized gender differences and social network characteristics in relation to physical activity during the COVID-19 pandemic [5,7], none have used mediation analysis to quantify the indirect effect of gender on physical activity through social networks.

Based on our mediation analyses, we found that the association between being female and having lower past-week physical activity was partially mediated by a key feature of online social network structure, i.e., the number of Facebook friends. The non-significant effects of friend network size and friend contact frequency (in-person) between gender and both past-week and past-year physical activity suggest that online social network characteristics may be more salient than in-person social networks for influencing women’s physical activity. Consistent with these findings, pre-pandemic studies found that women were more likely to use Facebook to maintain contact with a variety of friends [16,47] and to have more Facebook friends then men [18]. On the other hand, men may be more willing to seek new Facebook friends [16] and to have more intimate discussions with their new Facebook friends [48]. These gender-based variations in social network characteristics may influence gender disparities in physical activity during a pandemic. It is important to note that a higher number of Facebook friends was significantly inversely associated with lower levels of past-week physical activity. It is possible that the inverse relationship between Facebook friends and physical activity may be explained by the fact that women spend more time on Facebook then men [20,49] and are more likely to be habituated to Facebook then men [20]. Thus, as women try to maintain a large network of Facebook friends, they may have less time available to exercise due to the extra time spent on Facebook relative to men.

The results of this study are consistent with other research supporting the use of media technologies, such as social media platforms, to encourage physical activity during the COVID-19 pandemic [50,51]. Our findings add new understanding on using online social networks (i.e., Facebook friends) to promote physical activity among women during a pandemic. Future research should further examine the use of media technology via other social networking sites (e.g., Twitter) to help encourage physical activity among women during a pandemic or other natural disasters.

## 5. Implications

Findings suggest that social media sites such as Facebook could be used to facilitate and encourage physical activity among women during a pandemic. Specifically, pre-pandemic, women’s high number of Facebook friends were used to support online workouts, to motivate and encourage women to exercise, and to serve as accountability partners through observing/tracking group-based activity via mobile apps [14,52]. Thus, while women’s current Facebook networks may emphasize sedentary behaviors, it may be possible to harness existing Facebook networks to help women transition to more active lifestyles during a pandemic.

Because this study took place during a pandemic, it is unclear if findings would be replicated in non-pandemic times, as there is a lack of pre-pandemic data on the mediating effect of social networks in explaining gender-based differences in physical activity. However, as COVID-19 becomes endemic, there are likely to be periods with higher and lower rates of COVID [53], and these natural variations in COVID rates will provide further opportunities to test the limits of generalizability. The current study, which used reproducible measures via a popular online data collection platform, can serve as an initial step in clarifying the influence of social networks on gender disparities in physical activity and may help set the stage for further confirmation in other independent samples.

### Limitations

This cross-sectional study included a relatively small, U.S. convenience sample of mostly white, healthier adults, which limits causal inferences and generalizability to more diverse populations. This study only included male and female participants; thus, we did not consider non-binary gender status in our analysis. Additionally, other factors, such as mental illness (e.g., anxiety) [5] and sleep quality [54] that could influence social networks and physical activity during a pandemic, were not examined, which may have affected the results. The study also did not assess multiple types of online social network influences that may impact physical activity, such as use of Instagram, Twitter, LinkedIn, or other social networking sites. Furthermore, participants’ frequency of interacting with their Facebook friends, and the characteristics of those interactions, along with overall time spent on social media, were not captured. Thus, the actual mechanism by which Facebook friends influence physical activity still warrants further investigation. Moreover, mediator analyses emphasized a single dimension of the social environment (i.e., network size), and our analyses did not incorporate other potential mediator variables, such as extraversion [55] or enjoyment [56] of physical activity. Furthermore, although the past-week and past-year physical activity questionnaires exhibit evidence of reliability and validity [22,23,24,32], it is possible that responses to these questionnaires may be subject to recall biases or measurement error. Finally, we did not include a control group to assess pre-pandemic activity levels and social network characteristics.

## 6. Conclusions

Despite these limitations, our findings contribute to understanding the observed gender gap in physical activity during a pandemic. The identification of Facebook friends number as a mediator between gender and physical activity suggests that this social network characteristic may warrant further examination to help women adopt more active lifestyles during a pandemic. Future studies should explore how the online social media environment can be harnessed across the lifespan, and among diverse communities, to address gender inequality in physical activity during pandemic times and other situations associated with social isolation.

## Figures and Tables

**Table 1:** Characteristics of the study sample (N = 205).

Variable		n (%)
Gender ^a^	Female	129 (62.93)
	Male	76 (37.07)
Ethnicity ^b^	Not Hispanic or Latino	198 (96.59)
	Hispanic or Latino	7 (3.41)
Race ^c^	American Indian or Alaskan Native	2 (.98)
	Asian	14 (6.83)
	Black or African American	14 (6.83)
	White	175 (85.36)
Education ^d^	High school or GED	25 (12.19)
	Some college	40 (19.51)
	2-year college degree	29 (14.15)
	4-year college degree	71 (34.63)
	Master’s degree	31 (15.12)
	Doctoral degree	9 (4.39)
Marital status ^e^	Single and never married	66 (32.20)
	Married	96 (46.83)
	Divorced	22 (10.73)
	Separated	4 (1.95)
	Widowed	2 (.98)
	Living with partner	15 (7.32)
Income ^f^	Less than $30,000	46 (22.44)
	$30,000–$49,999	53 (25.85)
	$50,000–$69,999	40 (19.51)
	$70,000–$89,999	25 (12.19)
	$90,000–$109,999	18 (8.78)
	$110,000–$149,999	16 (7.80)
	$150,000 or greater	7 (3.41)
Residence ^g^	Single-family house	154 (75.12)
	Multi-family house	3 (1.46)
	Apartment	25 (12.20)
	Condominium	4 (1.95)
	Townhouse	10 (4.88)
	Duplex	6 (2.93)
	Other	3 (1.46)
Health Status ^h^	Excellent	38 (18.54)
	Very Good	73 (35.61)
	Good	61 (29.76)
	Fair	30 (14.63)
	Poor	3 (1.46)
		Mean (SD)
Age		40.98 (12.89)
Average number of Facebook friends		255.81 (375.37)
Average moderate-vigorous physical activity (NHIS)		168.79 (220.23)
Average moderate-vigorous physical activity per week over the past year (CT-PAQ)		232.27 (350.64)

Note: ^a^ Gender was coded as 0 = male and 1 = female; ^b^ Ethnicity was coded as 0 = Not Hispanic or Latino and 1 = Hispanic or Latino; ^c^ Race was coded as 1 = American Indian or Alaskan Native and 4 = White; ^d^ Education was coded as 1 = High school or GED and 6 = Doctoral degree; ^e^ Marital status was coded as 1 = Single and never married and 6 = Living with partner; ^f^ Income was coded as 1 = Less than $30,000 and 7 = $150,000 or greater; ^g^ Residence was coded as 1 = Single-family house and 7 = Other; ^h^ Health status was coded as 1 = Excellent and 5 = Poor.

**Table 2:** Indirect effects of mediator variables of gender on past-week physical activity.

Pathway	Indirect Effect			
	β	SE	Bootstrapped 95% CI	
			Lower	Upper
Gender—All Mediator Variables—Physical Activity	−24.88	12.70	−50.29	−4.07
Gender—Friend Network Size—Physical Activity	−3.39	6.00	−18.24	5.90
Gender—Friend Contact Frequency—Physical Activity	−2.36	5.28	−15.66	6.63
Gender—Facebook Friends—Physical Activity	−19.13	9.71	−40.45	−2.09

Notes: Dependent Variable: NHIS Moderate-Vigorous Minutes (past-week physical activity).

## Data Availability

The data presented in this study are available on request from the corresponding author.

## Supplementary Materials

The following supporting information can be downloaded at: https://www.mdpi.com/article/10.3390/ijerph19052495/s1, Syntax S1: SPSS syntax that was written to screen for attention check questions.

## Abbreviations

CDC	Centers for Disease Control and Prevention
CI	Confidence Interval
CT-PAQ	Chasan-Taber Physical Activity Questionnaire
HIT	Human Intelligence Task
ID	Identification
MTurk	Mechanical Turk
NHIS	National Interview Survey
SD	Standard Deviation
SNAP	Social Networks for Activity Promotion
SNI	Berkman–Syme Social Network Index
US	United States
