# Supplementary material for: The Association between Gender and Physical Activity Was Partially Mediated by Social Network Size during COVID-19
Source: Int J Environ Res Public Health. 2022 Feb 22;19(5):2495. doi: 10.3390/ijerph19052495 (PMC8909104; doi:10.3390/ijerph19052495)
Supplement: Supplementary file 1 [file ijerph-19-02495-s001.zip › ijerph-1565141-supplementary.pdf]

Supplementary Materials
Syntax S1: SPSS syntax that was written to screen for attention check questions.

```
COMPUTE workidAgree_ELIG=survey1_workid = screen_workid.
EXECUTE.

COMPUTE YearBornApproxAge=2020 - yearborn.
EXECUTE.

COMPUTE AgeDiscrepancyAmt=YearBornApproxAge - age.
EXECUTE.

RECODE AgeDiscrepancyAmt (1=1) (-1=1) (0=1) (ELSE=0) INTO AgeDiscrep_ELIG.
VARIABLE LABELS  AgeDiscrep_ELIG 'Eligible to continue based on accuracy of age estimation'.
EXECUTE.

COMPUTE WorkIDAgree_FinELIG=workidAgree_ELIG + 0.
EXECUTE.

COMPUTE AgeDiscrep_FinELIG=AgeDiscrep_ELIG + 0.
EXECUTE.

COMPUTE SumEligibility=SUM(WorkIDAgree_FinELIG,AgeDiscrep_FinELIG)
EXECUTE.

RECODE SumEligibility (2=1) (ELSE=0) INTO ELIGFINAL_Survey1.
VARIABLE LABELS ELIGFINAL_Survey1 'SumEligibility recoded'.
EXECUTE.
```
